# No Association of PTPN22 Polymorphisms with Susceptibility to Ocular Behcet's Disease in Two Chinese Han Populations

**DOI:** 10.1371/journal.pone.0031230

**Published:** 2012-03-02

**Authors:** Qi Zhang, Shengping Hou, Zhengxuan Jiang, Liping Du, Fuzhen Li, Xiang Xiao, Aize Kijlstra, Peizeng Yang

**Affiliations:** 1 Chongqing Key Laboratory of Ophthalmology and Chongqing Eye Institute, The First Affiliated Hospital of Chongqing Medical University, Chongqing, People's Republic of China; 2 Department of Ophthalmology, Eye Research Institute Maastricht, University Hospital Maastricht, Maastricht, The Netherlands; IFOM, Fondazione Istituto FIRC di Oncologia Molecolare, Italy

## Abstract

**Background:**

Behcet's disease is known as a recurrent, multisystem inflammation and immune-related disease. Protein tyrosine phosphatase non-receptor 22 (*PTPN22*) is a key negative regulator of T lymphocytes and polymorphisms of the *PTPN22* gene have been shown to be associated with various immune-related diseases. The present study was performed to assess the association between *PTPN22* polymorphisms and Behcet's disease in two Chinese Han populations.

**Methodology/Principal Findings:**

A total of 516 patients with ocular Behcet's disease and 690 healthy controls from two Chinese Han populations were genotyped by the polymerase chain reaction-restriction fragment length polymorphism (PCR-RFLP) method for three single nucleotide polymorphisms (SNPs). Hardy-Weinberg equilibrium was tested using the χ^2^ test. Genotype frequencies were estimated through direct counting. Allele and genotype frequencies were compared between patients and controls using logistic regression analysis. The results revealed that there was no association between the tested three *PTPN22* SNPs (rs2488457, rs1310182 and rs3789604) and ocular Behcet's disease (*p*>0.05). Categorization analysis according to the clinical features did not show any association of these three polymorphisms with these parameters (*p*>0.05).

**Conclusions/Significance:**

The investigated *PTPN22* gene polymorphisms (rs2488457, rs1310182 and rs3789604) were not associated with ocular Behcet's disease in two Chinese Han populations, and showed that it may be different from other classical autoimmune diseases. More studies are needed to confirm these findings for Behcet's disease in other ethnic backgrounds.

## Introduction

Behcet's disease (BD) is known as a multisystem inflammatory disorder of unknown etiology. It is characterized by recurrent attacks of uveitis, oral ulceration, genital ulceration, skin lesions, arthritis, and vascular inflammation [1]. It is curently recognized as an immune-related disease presenting with signs of immune hyper-reactivity mainly affecting elements of the innate immune system. Although the precise triggering factors for BD are still unclear, complex mechanisms including genetic, environmental and immunologic, are presumed to be involved [2]–[5]. Genetic susceptibility to BD has been investigated in different populations throughout the world. Human leukocyte antigen (*HLA*)-*B51* is thought to be the most strongly associated genetic factor predisposing to BD. The contribution of *HLA*-*B51* to the genetic risk of BD has however been estimated to be less than 20%. Therefore study on non-*HLA* genes, especially related to the immune response and inflammation is of great importance. Several immune relevant genes, such as the *IL-23* receptor gene, *IL-10* gene and *SUMO4* gene, have been revealed to be risk factors for BD [6]–[9].

The protein tyrosine phosphatase non-receptor 22 (*PTPN22*) gene maps to chromosome 1p13.3-p13.1, and encodes the lymphoid-specific phosphatase known as Lyp, which contains a catalytic N-terminal domain and a non-catalytic C-terminus composed of four proline-rich domains. Lyp is an important down-regulator of T-cell activation through interacting with protein tyrosine kinase (Csk) and inhibiting signaling pathways mediated by the T-cell receptor (TCR) [10]. Several studies have revealed that the nucleotide diversity of *PTPN22* could trigger the immune and inflammation response by decreasing the affinity between Lyp and Csk, thereby inhibiting the TCR signaling pathway [11]–[13]. *PTPN22* has been shown to be associated with susceptibility to a number of immune related-diseases, such as type 1 diabetes (T1D), rheumatoid arthritis (RA) and systemic lupus erythematosus (SLE) [14]–[16].

Earlier studies in a Turkish population with BD analyzed the rs2476601 genotype of the *PTPN22* gene and concluded that this polymorphism had a limited (protective) role in disease pathogenesis [17]. A similar study including UK and Middle-East patients with BD showed a protective effect of the T1858 allele in the Middle East patients but not in the UK group [18]. As yet, in Chinese Han populations, no data are available concerning a possible association of *PTPN22* gene with BD, and this was therefore the purpose of the study presented here. The frequencies of some alleles display great variation in different regions and ethnic groups worldwide. The International HapMap data and recent reports showed the SNP rs2476601 (1858C/T) are not polymorphic in Chinese Han populations, although they have been shown to be associated with susceptibility for immune diseases in other ethnic groups [19]–[21]. In Asian populations, other candidate SNPs of this gene, namely rs2488457, rs1310182 and rs3789604, were confirmed to be associated with several immune diseases, such as T1D, RA and Graves disease (GD) [22]–[26]. In view of these earlier findings we limited our analysis to these three SNPs (rs2488457, rs1310182 and rs3789604). The results failed to show an association of *PTPN22* gene polymorphisms with risk to ocular BD.

## Results and Discussion

No statistical differences were observed in the distribution of age for the Chinese Han case-control cohorts (*p*>0.05). There were also no significant differences in the distribution of gender for the case-control study in groups (*p*>0.05). The age and gender distribution of the ocular BD patients and healthy controls are shown in Table 1. The clinical features of the ocular BD patients included in our study are presented in Table 2.

**Table 1:** Characteristics of the investigated ocular BD patients and healthy controls.

Characteristics	Cohort1			Cohort2			Total		
	BD (n = 251)	Controls (n = 336)	P value	BD (n = 265)	Controls (n = 354)	P value	BD (n = 516)	Controls (n = 690)	P value
Age(y) mean±SD	31.2±6.7	32.0±7.1	0.167	32.5±7.1	33.4±7.8	0.141	32.3±7.5	32.9±8.0	0.186
Age(y) median	32.0	36.0		34.0	34.0		33.0	35.0	
Age(y) range	13–60	17–72		10–67	12–71		10–67	12–72	
Male n (%)	206(82.1)	274(81.5)	0.871	223(84.2)	296(83.6)	0.858	429(83.1)	570(82.6)	0.809
Female n (%)	45(17.9)	62(18.5)		42(15.8)	58(16.4)		87(16.9)	120(17.4)	

**Table 2:** Clinical features of the investigated ocular BD patients.

Clinical features	patients with Behcet's disease	
	n (total)	%
Uveitis	516	100
Oral ulcer	498	96.5
Genital ulcer	189	36.6
Hypopyon	117	22.7
Skin lesions	241	46.7
Positive pathergy test	176	34.1
Arthritis	133	25.8

A total of 516 ocular BD patients and 690 healthy controls were genotyped for three SNPs of *PTPN22* (rs2488457, rs1310182 and rs3789604). The RFLP genotyping matched results obtained by direct sequencing. The genotype and allele frequencies of the three SNPs of *PTPN22* examined in BD patients and healthy controls are summarized in Tables 3, 4, 5. The results showed that the distribution of genotypes and alleles did not deviate from the Hardy–Weinberg equilibrium (HWE). There were no differences concerning the genotype or allele frequencies of the three SNPs between ocular BD patients and healthy controls respectively in the two cohorts (*p*>0.05), and similar results were observed when comparing the combined patient cohorts with the controls (*p*>0.05). Under the null hypothesis (absence of association between disease and genotype/allele), OR (odd ratio) is expected to be 1.000.

**Table 3:** Genotype and allele frequencies of *PTPN22* polymorphisms between the ocular BD patients and healthy controls in Guangzhou.

SNP	Genotype Allele	BD n (%)	Controls n (%)	P value	OR (95%CI)
rs2488457	GG	42(16.7)	47(14.0)		1.000
	CG	124(49.4)	187(55.7)	0.217	0.742(0.462–1.192)
	CC	85(33.9)	102(30.3)	0.787	0.933(0.562–1.547)
	G	208(41.4)	281(41.8)		1.000
	C	294(58.6)	391(58.2)	0.896	1.016(0.803–1.284)
rs1310182	CC	14(5.6)	17(5.0)		1.000
	CT	73(29.1)	97(28.9)	0.817	0.913(0.423–1.972)
	TT	164(65.3)	222(66.1)	0.771	0.896(0.430–1.871)
	C	101(20.1)	131(19.5)		1.000
	T	401(79.9)	541(80.5)	0.79	0.961(0.719–1.285)
rs3789604	TT	150(59.8)	208(61.9)		1.000
	GT	96(38.2)	120(35.7)	0.551	1.109(0.789–1.560)
	GG	5(2.0)	8(2.4)	0.805	0.867(0.278–2.701)
	T	396(78.9)	536(79.8)		1.000
	G	106(21.1)	136(20.2)	0.713	1.055(0.793–1.403)

OR = odds ratio; 95%CI = 95% confidence interval.

The number of BD patients was 251, and the number of controls was 336.

**Table 4:** Genotype and allele frequencies of *PTPN22* polymorphisms between the ocular BD patients and healthy controls in Chongqing.

SNP	Genotype Allele	BD n (%)	Controls n (%)	P value	OR (95%CI)
rs2488457	GG	33(12.5)	40(11.3)		1.000
	CG	126(47.5)	189(53.4)	0.416	0.808(0.484–1.350)
	CC	106(40.0)	125(35.3)	0.919	1.028(0.606–1.744)
	G	192(36.2)	269(38.0)		1.000
	C	338(63.8)	439(62.0)	0.525	1.079(0.854–1.362)
rs1310182	CC	9(3.4)	11(3.1)		1.000
	CT	70(26.4)	92(26.0)	0.878	0.929(0.365–2.366)
	TT	186(70.2)	251(70.9)	0.829	0.905(0.368–2.229)
	C	88(16.6)	114(16.1)		1.000
	T	442(83.4)	594(83.9)	0.813	0.964(0.711–1.307)
rs3789604	TT	163(61.5)	230(65.0)		1.000
	GT	94(35.5)	114(32.2)	0.381	1.163(0.829–1.633)
	GG	8(3.0)	10(2.8)	0.803	1.129(0.436–2.922)
	T	420(79.2)	574(81.1)		1.000
	G	110(20.8)	134(18.9)	0.424	1.122(0.846–1.487)

OR = odds ratio; 95%CI = 95% confidence interval.

The number of BD patients was 265, and the number of controls was 354.

**Table 5:** Genotype and allele frequencies of *PTPN22* polymorphisms between the combined group of ocular BD patients and healthy controls.

SNP	Genotype Allele	BD n (%)	Controls n (%)	P value	OR (95%CI)
rs2488457	GG	75 (14.5)	87 (12.6)		1.000
	CG	250(48.5)	376(54.5)	0.143	0.771(0.545–1.092)
	CC	191(37.0)	227(32.9)	0.896	0.976(0.678–1.404)
	G	400(38.8)	550(39.9)		1.000
	C	632(61.2)	830(60.1)	0.586	1.047(0.888–1.235)
rs1310182	CC	23 (4.5)	28 (4.1)		1.000
	CT	143(27.7)	189(27.4)	0.784	0.921(0.509–1.665)
	TT	350(67.8)	473(68.5)	0.717	0.900(0.510–1.590)
	C	189(18.3)	245(17.8)		1.000
	T	843(81.7)	1135(82.2)	0.723	0.963(0.781–1.187)
rs3789604	TT	313(60.7)	438(63.5)		1.000
	GT	190(36.8)	234(33.9)	0.297	1.136(0.894–1.445)
	GG	13 (2.5)	18 (2.6)	0.977	1.011(0.488–2.093)
	T	816(79.1)	1110(80.4)		1.000
	G	216(20.9)	270(19.6)	0.407	1.089(0.891–1.330)

OR = odds ratio; 95%CI = 95% confidence interval.

The number of BD patients was 516, and the number of controls was 690.

In addition, we investigated whether the *PTPN22* SNPs were associated with the various clinical features of ocular BD, such as oral ulceration, genital ulceration, hypopyon, skin lesions, positive pathergy test and arthritis. The analysis did not indicate any significant association of these parameters with the tested three SNPs of *PTPN22* (*p*>0.05) (data not shown).

Numerous factors have been reported to influence the results of the study on the association of gene polymorphisms with disease. We made the following efforts to ensure correctness of the results. The ocular BD patients were selected strictly according to the criteria of the International Study Group or the revised criteria from Behcet's Disease Research Committee of Japan if oral ulceration was not present, and two cohorts of the samples came from two independent uveitis centers in China. The healthy individual controls were recruited from the same geographical regions as the patients. No statistical differences were observed in the distribution of age and gender for the collected Chinese Han case-control cohorts. In order to validate the results of genotyping by PCR-restriction fragment length polymorphism, 10% of the samples were randomly chosen and confirmed by direct sequencing.

During recent years the *PTPN22* gene has emerged as an important candidate susceptibility factor for a number of immune diseases [27]. A role of *PTPN22* in clinical uveitis had been addressed in British, Turkish and Middle-East BD patients and described a limited (protective) effect of the T1858 allele in the Middle East patients but not in the UK group [17], [18]. Similarly, Martin *et al* showed no association between the R620W (rs2476601) of *PTPN22*, 1858C/T genotype and acute anterior uveitis [28]. Wagenleiter *et al* revealed that the above polymorphism of *PTPN22* gene had no influence on Crohn's disease (CD) in Germans [29]. Matesanz *et al* also failed to demonstrate any correlation of the above SNP with Multiple sclerosis (MS) in Spanish patients [30]. On the other hand, an association with *PTPN22* polymorphisms has consistently been reported as a susceptibility gene for a number of autoimmune diseases characterized by specific autoantibodies [20]. These studies suggest that BD, CD and MS may have a different genetic background and pathogenesis as compared with classical antibody mediated autoimmune diseases such as T1D, RA and SLE. On the other hand, autoimmunity in BD is still a controversial issue. Numerous studies suggest that BD may be mainly prone to autoinflammatory processes as opposed to autoimmunity [17], [18], [31]–[33]. Since *PTPN22* has always been presumed to be a genetic switch for autoimmunity, the lack of an association with the common SNPs of this gene may provide additional evidence to the hypothesis that BD is autoinflammatory rather than an autoimmune disease.

Like other candidate gene research, there are some limitations in our study. The size of patient samples seems to be relatively small if the pathogenic role of the *PTPN22* gene is not strong enough. The patients were only recruited from the Chinese Han population. Therefore, the association of *PTPN22* polymorphisms with BD should be studied using a larger sample size and other ethnic populations. On the other hand, BD is a complex disease that affects multiple systems, and the tested patients from an ophthalmology department represent a subpopulation of this disease. The association of *PTPN22* polymorphisms with BD should therefore also be investigated in BD patients from other medical departments, such as dermatology and stomatology. Finally, the present study only examined three of the *PTPN22* polymorphisms, therefore it does not exclude the possibility that other SNPs of *PTPN22* are associated with BD. More studies are needed to clarify these issues.

In conclusion, our study failed to find any association between the three polymorphisms of *PTPN22* gene (rs2488457, rs1310182 and rs3789604) and susceptibility for ocular BD in Chinese Han patients.

## Materials and Methods

### Clinical Samples

Two independent Chinese Han population case-control cohorts were included in this study. Cohort 1 consisted of 251 ocular BD cases and 336 controls collected at the Zhongshan Ophthalmic Center (Guangzhou, P.R. China) from January 2005 to March 2008. Cohort 2 consisted of 265 ocular BD cases and 354 controls collected at the First Affiliated Hospital of Chongqing Medical University (Chongqing, P.R. China) from March January 2008 to May 2011. All patients had intraocular inflammation. In total, there were 516 patients with ocular BD and 690 healthy controls, whereby the comparison was performed as a case-control study in groups. Controls were not matched to case, individually or in larger groups. The proportion of male vs female is 5∶1 to 4∶1 both in the cases and controls. The diagnosis of BD was made according to the criteria of the International Study Group for BD or the revised criteria from Behcet's Disease Research Committee of Japan if oral ulceration was not present [34], [35]. The clinical characteristics of BD patients were assessed at the time of diagnosis.

### Ethics statement

The protocol was approved by the Ethics Committee of the First Affiliated Hospital of Chongqing Medical University, Chongqing, China (Permit Number: 2009-201004), and written informed consent was obtained from all the study subjects. All procedures followed the tenets of the Declaration of Helsinki.

### Genomic DNA extraction and genotyping

Blood samples were collected in EDTA tubes and kept at −70°C until used. Genomic DNA was extracted by the QIAamp DNA Blood Mini Kit (Qiagen, Hilden, Germany). Amplification of the target DNA in the PTPN22 gene was analyzed by the polymerase chain reaction (PCR) using primers presented in Table 6. Each PCR reaction was performed in 10 µl containing 5 µl Premix Taq (Promega, Madison, USA), 20 pmoles primers and 0.2 µg of genomic DNA. The PCR conditions were as follow: initial denaturation at 95°C for 5 minutes followed by 37 cycles of denaturation at 94°C for 30 seconds, annealing at different temperatures (58°C for rs2488457, 58°C for rs1310182, and 60°C for rs3789604) for 30 seconds, extension at 72°C for 30 seconds, and a final extension at 72°C for 5 minutes. These SNPs were genotyped by PCR-restriction fragment length polymorphism (RFLP) analysis. PCR products of rs2488457, rs1310182 and rs3789604 polymorphisms were respectively digested with 4 U of MnlI (MBI Fermentas, Vilnius, Lithuania), Hsp92II (Promega, Madison, USA), and BseXI (MBI Fermentas, Vilnius, Lithuania) restriction enzymes (Table 6) in a 10 µl reaction volume overnight. Digestion products were visualized on a 3.5% agarose gel and stained with GoldView (SBS Genetech, Beijing, China). Randomly selected subjects (10% of all samples) were directly sequenced (Invitrogen Biotechnology, Shanghai, China) to validate the polymerase chain reaction restriction fragment length polymorphism (PCR-RFLP) results.

**Table 6:** Primers and restriction enzymes used for RFLP analysis of *PTPN22*.

rs number	Primers	Restriction enzyme
rs2488457	5′-GTACCCATTGAGAGGTTATGCAACCT-3′	MnlI
	5′-TGTTTCAGGAAGTTATGCTGTGCC-3′	
rs1310182	5′-AAACCCAATGACCAATGACA-3′	Hsp92II
	5′-AAGCATTTAATTATATGGTGCTGAG-3′	
rs3789604	5′-TTTAAATGGCCCGACCGCCCCCCCGT-3′	BseXI
	5′-TGGGAACGGCCGCTTCCGGCATG-3′	

### Statistical methods

Hardy-Weinberg equilibrium was tested using the χ^2^ test. The variable age, described as mean ± standard deviation (SD), median and range, between cases and controls was compared using the student's t-test. The variable gender was compared with Chi-square tests between cases and controls. Genotype frequencies were estimated by direct counting. Allele and genotype frequencies were compared between patients and controls by the unconditional logistic regression analysis (SAS, 9.13), which was used to compute the odds ratios (ORs) and their 95% confidence intervals (CIs). This statistical method was also performed to analyze the association between the SNPs and various clinical features of ocular BD. All statistical tests were two-sided. P<0.05 was considered significant.
